# Towards Point of Care CRISPR-Based Diagnostics: From Method to Device

**DOI:** 10.3390/jfb14020097

**Published:** 2023-02-10

**Authors:** Haoxiang Chen, Xi Zhou, Miao Wang, Lei Ren

**Affiliations:** 1 The Higher Educational Key Laboratory for Biomedical Engineering of Fujian Province, Research Center of Biomedical Engineering of Xiamen, Department of Biomaterials, College of Materials, Xiamen University, Xiamen 361005, China; 2 State Key Lab of Physical Chemistry of Solid Surfaces, Xiamen University, Xiamen 361005, China

**Keywords:** CRISPR/Cas, molecular diagnostic, point of care, DNA detection

## Abstract

Rapid, accurate, and portable on-site detection is critical in the face of public health emergencies. Infectious disease control and public health emergency policymaking can both be aided by effective and trustworthy point of care tests (POCT). A very promising POCT method appears to be the clustered regularly interspaced short palindromic repeats and associated protein (CRISPR/Cas)-based molecular diagnosis. For on-site detection, CRISPR/Cas-based detection can be combined with multiple signal sensing methods and integrated into smart devices. In this review, sensing methods for CRISPR/Cas-based diagnostics are introduced and the advanced strategies and recent advances in CRISPR/Cas-based POCT are reviewed. Finally, the future perspectives of CRISPR and POCT are summarized and prospected.

## 1. Introduction

Nucleic acid-based tests have emerged as the gold standard for many diseases, including pathogenic infections, despite the fact that there are various forms of clinical diagnosis. Quantitative polymerase chain reaction (qPCR)-based nucleic acid testing is also widely employed in many fields including public health, food safety, environmental monitoring, etc., [1,2]. Because this method relies on specialist instruments and operators, point of care testing (POCT), which is instrument-independent and user-friendly, has received a lot of attention, especially during the global SARS-CoV-2 pandemic. In recent years, numerous technologies have been developed and applied to molecular diagnostics, such as nanotechnology, nucleic acid amplification, interface modification and sensing, and molecular assembly techniques [3,4,5]. In particular, CRISPR-based diagnostics have been hailed as a promising candidate for next-generation diagnostics because of their capacity to detect nucleic acids rapidly and accurately, without relying on technical expertise and assistive equipment [6].

CRISPR/Cas systems are the adaptive immune system of bacteria, which can resist the invasion of exogenous genes [7,8]. CRISPR/Cas systems consist of the CRISPR array and CRISPR-associated protein (Cas protein). The CRISPR array contains repeats and spacer sequences to generate CRISPR RNA (crRNA), which combines with the corresponding Cas protein to form a ribonucleoprotein (RNP) complex that functions as a genomic DNA editor [9]. CRISPR/Cas systems have been divided into two classes, and class 2 systems are widely used because they require only one Cas protein (e.g., Cas9, Cas12a, Cas13a) to fulfill their corresponding functions [10,11]. Since the team led by Emmanuelle Charpentier and Jennifer A. Doudna clarified the DNA-editing mechanism of the class 2 CRISPR system, genome engineering has gained tremendous momentum [8,11]. With the comprehensive understanding of CRISPR/Cas biology, the indiscriminate trans-cleavage activity of Cas12a and Cas13a has been reported and can be used in combination with single-strand DNA (ssDNA) or single-strand RNA (ssRNA) probes for the development of molecular diagnostic methods [12,13,14]. After target cleavage, the Cas9 protein goes into an inactive state, but when the Cas12a and Cas13a proteins are activated, indiscriminate trans-cleavage activity is maintained, in stark contrast to Cas9. Due to this unique property, both the Cas12a and Cas13a proteins can be triggered by a single target sequence to acquire multiple nuclease activities. These activities significantly increase sensitivity by acting as superior signal amplifiers for nucleic acid detection. Furthermore, this reaction procedure can be carried out speedily under room temperature, making it ideal for developing the CRISPR-based POCT technology platform, and for use in clinical and field settings.

The powerful nucleic acid binding and broader cleavage capabilities of Cas proteins lead to several detection modes, including fluorescence, electricity, electrochemistry, colorimetry, and more [15,16,17,18]. However, the CRISPR-based POCT technique that satisfies the ASSURED criteria (Affordable, Sensitive, Specific, User-friendly, Rapid and robust, Equipment-free, and Deliverable to end-users) is probably going to be a major research trend in the future to improve potential for public health applications of CRISPR-based biosensing [19,20]. In this review, we briefly summarize CRISPR-based sensing methods and advanced strategies for the highly sensitive and accurate detection of different analytes. Although CRISPR-based diagnostics have considerable obstacles in reducing uptime and cost, advancements in these strategies hold promise for adapting CRISPR to point of care and home settings.

## 2. Sensing Methods for CRISPR/Cas-Based Detection

### 2.1. Fluorescence Signal Sensing

**Figure 1:**
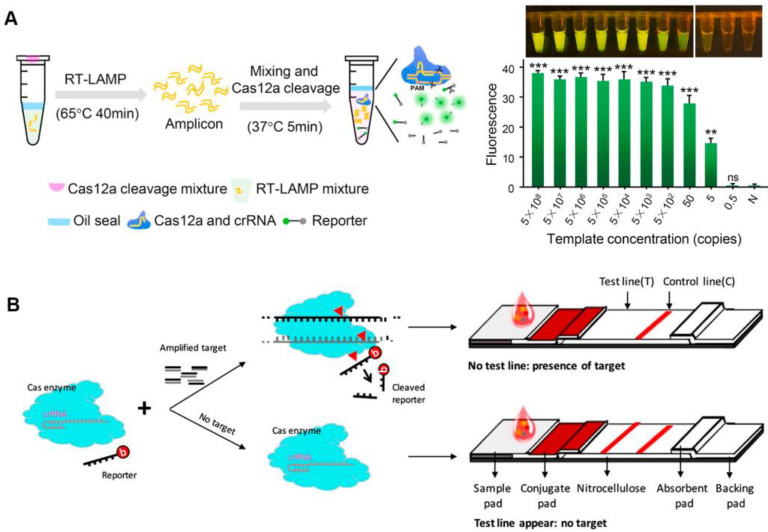
Fluorescence sensing strategy for CRISPR-based diagnosis. (**A**) The scheme of one-pot visual CRISPR-based (opvCRISPR) detection method. (ns = no significant, ** *p* < 0.01, *** *p* < 0.001) Reproduced with permission from Ref. [25]. (**B**) Schematic diagram of CRISPR/Cas-based lateral flow assay. Reproduced with permission from Ref. [31].

The variety of nucleic acids that can be detected have been enriched by the discovery of Cas proteins with various properties (Table 1). After activation, both Cas12a and Cas13a proteins show a trans-cleavage activity and can cleave single-stranded DNA (ssDNA) and single-stranded RNA (ssRNA) nonspecifically, respectively [12,13]. Upon the recognition of the target sequence by Cas12a or Cas13a, the Cas effector proteins can indiscriminately cleave short ssDNA or ssRNA bearing both a fluorophore and a quencher, reporting a fluorescent signal [21,22]. CRISPR-based nucleic acid diagnosis is usually divided into four steps. Firstly, nucleic acid extraction. Commercial kits can be used to swiftly extract the target nucleic acid in clinical samples such as blood, urine, and nose/throat swabs, etc. Secondly, nucleic acid amplification. Isothermal amplification is frequently used for the second step. Thirdly, CRISPR-based detection, with CRISPR system selection according to different nucleic acid types. Finally, signal output. Fluorescence signals are common CRISPR sensing signals that facilitate analysis. The viral genomic nucleic acid detection limits of the specific high-sensitivity enzymatic reporter unlocking (SHERLOCK) and the DNA endonuclease-targeted CRISPR trans reporter (DETECTR) developed following this principle, are at the aM level [12,23]. CRISPR diagnosis based on fluorescence signal has great advantages in responding to public health emergencies (such as SARS-CoV-2), especially when combined with isothermal amplification technology, which can rapidly detect infectious disease pathogen nucleic acids under isothermal conditions, and only requires a portable light-emitting diode (LED) device to visualize detection results [24]. Meanwhile, isothermal amplification and CRISPR-based diagnostics can be integrated into one pot, avoiding the aerogel contamination of pathogenic nucleic acids, while also reducing detection costs (Figure 1A) [25,26]. The CRISPR detection outcomes based on fluorescence signals are generally simple to see with the naked eye, requiring no specialized analytical tools and significantly lowering the cost of CRISPR-based POCT.

To employ CRISPR/Cas-based diagnostics in the field, a lateral flow assay was combined with the CRISPR/Cas system to create a convenient detection platform [21,27]. Typically, carboxyfluorescein (FAM)-biotin reporters are used to carrying out lateral flow strip-based CRISPR/Cas12a detection assays. The first detection line (control-line) captures undegraded reporters, whereas the second detection line produces a signal from indiscriminate Cas12 trans-cleavage activity (test-line) [27]. Contrarily, in a different approach, the biotinylated ssDNA reporters are degraded by Cas12a in the presence of the target, making it invisible on the test-line of a lateral flow strip since it is unable to connect to DNA probes that have already been fixed there (Figure 1B). By using a one-pot method that combines genome release, isothermal amplification, and CRISPR/Cas detection, the nucleic acid detection of pathogens can be accomplished relatively quickly. The CRISPR/Cas-cleaved fluorescent molecules can be sensitively excited by the LED, enabling visualization of the detection signal. Meanwhile, this operationally friendly detection mode has been applied in a variety of pathogen detection, such as SARS-CoV-2, *Plasmodium*, and African swine-fever viruses, which makes it a convenient tool for public health detection in resource-constrained areas and on-site detection [28,29,30,31].

### 2.2. Electrical Signal Sensing

**Figure 2:**
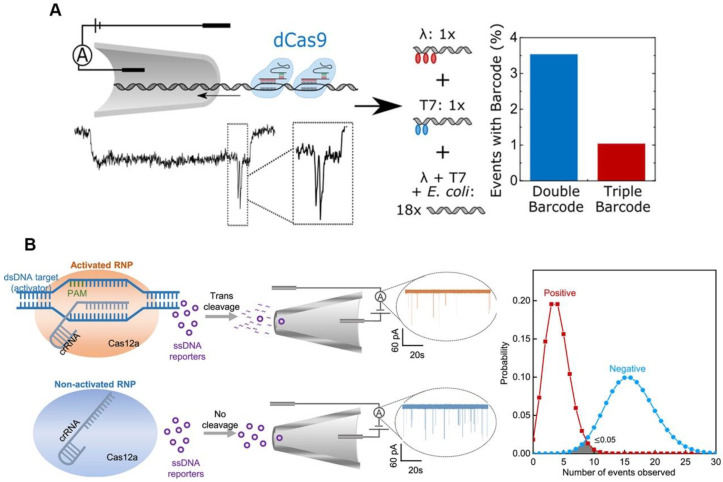
Electrical sensing strategy for CRISPR-based diagnosis. (**A**) Schematic diagram and results of nanopore-based CRISPR/dCas9 test. Reproduced with permission from Ref. [36]. (**B**) Schematic diagram and results of nanopore-based CRISPR/Cas12a assay. Reproduced with permission from Ref. [37].

The high sensitivity of CRISPR/Cas-based detection is typically based on isothermal amplification that can amplify nucleic acid molecules; however, the use of the isothermal amplification reagents raises the cost of CRISPR/Cas-based diagnosis. Because of its high sensitivity, electrical signal sensing could be a candidate for amplification-free CRISPR/Cas-based diagnosis [32,33]. Electrical signal-based CRISPR/Cas diagnosis is typically performed using a defective Cas protein such as dCas9, which can only bind and not cleave target nucleic acid molecules. Hajian et al. fixed the CRISPR/dCas9 to a field effect transistor (gFET), and when the RNP recognized and bound a target nucleic acid molecule, the gFET conductivity changed, therefore reporting the detection result [34]. Their sensor is based on the idea that charged DNA molecules can be captured by dCas9 immobilized on graphene, which lowers the system’s resistance and results in a larger current response. To prevent the nonspecific adsorption of charged molecules, the remaining graphene surface was blocked. A drop of the sample was applied to the apparatus while it was in use; it was left while it was incubated, washed, and eventually had the electrical conductivity of the platform evaluated by two platinum electrodes. The method has a detection limit of 15 fM without amplification, and the detection time is about 15 min. The nanopore sensor can also be used as a signal sensing method for CRISPR/dCas9-based diagnostic by detecting the characteristic blockade signal of dCas9-DNA as it passes through the nanopore [35]. The nanopore signals (ion current) for different dCas9-binding sites can be resolved, which could lead to a multiplexing strategy. Signal barcodes can be created to distinguish the different sequences of the DNA using different crRNAs, which can bind to different sites on the same DNA (Figure 2A) [36]. This method’s ability to discriminate between different DNA sequences in DNA mixture demonstrates its specificity and potential for multiplexing. Although dCas9 can detect and type single DNA, its application is limited by low throughput. Additionally, because there is a decreased probability of the target DNA strand passing through the nanopore in samples containing less target DNA, a longer measuring time is needed. The trans-cleavage of Cas12a may be able to provide a solution to this issue. The Cas12a trans-cleavage can degrade the DNA probes of nanopore, causing the electrical signal of the DNA probes to change significantly. The Cas12a-based nanopore sensing technology can amplify the detection signal, using its trans-cleavage activity to achieve rapid and sensitive detection of HIV-1 and SARS-CoV-2 (Figure 2B) [37,38].

### 2.3. Electrochemical Signal Sensing

**Figure 3:**
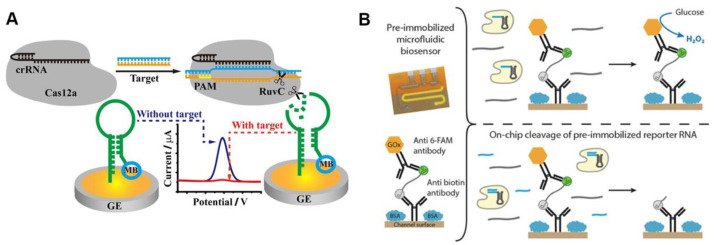
Electrochemical sensing for CRISPR-based diagnosis. (**A**) A schematic diagram of the CRISPR biosensing method based on electrochemical label. Reproduced with permission from Ref. [40]. (**B**) A working principle of glucose oxidase-based electrochemical CRISPR assay. Reproduced with permission from Ref. [42].

Methylene blue (MB) is a classic electrochemical tag, and the MB-based CRISPR diagnostic technique has high sensitivity. The ssDNA-MB was immobilized onto the gold electrode surface via sulfhydryl groups, and the MB was released when the ssDNA was trans-cleaved after the CRISPR/Cas12a was activated by the target sequences. The detection signal was reported using the current peak of the electrode measured by square wave voltammetry (SWV), which decreases with the release of MB [39]. The use of hairpin ssDNA allows MB tags to be positioned closer to the electrode surface, resulting in larger initial current peaks and more significant current peak changes after CRISPR/Cas12a-based detection (Figure 3A) [40]. The strategy of removing electrochemical tags from the electrode surface after the CRISPR/Cas system is activated leads to significant changes in the electrode’s electrochemical signal, which appears to be a promising route of CRISPR-based POCT [41]. 

The use of oxidative enzymes to amplify electrical signals appears to be a promising strategy. Bruch et al. immobilized glucose oxidase on the electrode surface using ssRNA, and the trans-cleavage activity of Cas13a cleaved ssRNA after being activated by the target sequence, releasing the glucose oxidase (Figure 3B). Glucose oxidase can oxidize the glucose in a solution to produce D-glucose-δ-lactones and H_2_O_2_, which can be measured amperometrically. The current signal will be lower than the blank if target RNA is present [42]. Additionally, independent of the electrochemical tag, the use of electrochemical impedance spectroscopy (EIS) as the detection signal may improve the electrochemical CRISPR-based diagnostic sensitivity. The electrode surface was modified with ssDNA, which was degraded after the CRISPR/Cas12a system recognized the target sequence, and the electrochemical impedance spectral transition of the electrode was reported as the detection signal [43]. In summary, the electrochemical CRISPR/Cas-based assay has achieved a highly sensitive detection of pathogens such as human papillomavirus 16 (HPV-16), human immunodeficiency virus (HIV), parvovirus B19 (PB-19), and *Listeria monocytogenes*, with great potential for POCT of infectious diseases [39,44,45]. Table 2 provides a summary of the performance of the electrochemical sensing-based CRISPR detection method described previously. Overall, the electrochemical sensing technique has a high sensitivity, allowing CRISPR detection without nucleic acid amplification.

The use of oxidative enzymes to amplify electrical signals appears to be a promising strategy. Bruch et al. immobilized glucose oxidase on the electrode surface using ssRNA, and the trans-cleavage activity of Cas13a cleaved ssRNA after being activated by the target sequence, releasing the glucose oxidase (Figure 3B). Glucose oxidase can oxidize the glucose in a solution to produce D-glucose-δ-lactones and H_2_O_2_, which can be measured amperometrically. The current signal will be lower than the blank if target RNA is present [42]. Additionally, independent of the electrochemical tag, the use of electrochemical impedance spectroscopy (EIS) as the detection signal may improve the electrochemical CRISPR-based diagnostic sensitivity. The electrode surface was modified with ssDNA, which was degraded after the CRISPR/Cas12a system recognized the target sequence, and the electrochemical impedance spectral transition of the electrode was reported as the detection signal [43]. In summary, the electrochemical CRISPR/Cas-based assay has achieved a highly sensitive detection of pathogens such as human papillomavirus 16 (HPV-16), human immunodeficiency virus (HIV), parvovirus B19 (PB-19), and *Listeria monocytogenes*, with great potential for POCT of infectious diseases [39,44,45]. Table 2 provides a summary of the performance of the electrochemical sensing-based CRISPR detection method described previously. Overall, the electrochemical sensing technique has a high sensitivity, allowing CRISPR detection without nucleic acid amplification.

### 2.4. Colorimetric Signal Sensing

**Figure 4:**
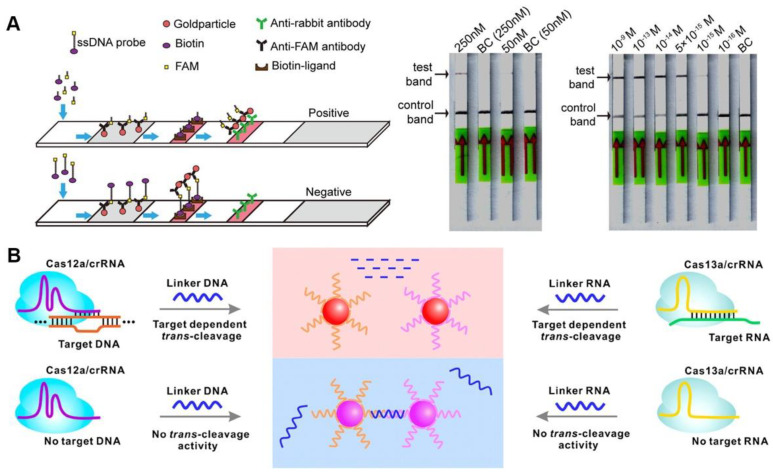
CRISPR-based colorimetric detection. (**A**) A schematic diagram and results of CORDS test. Reproduced with permission from Ref. [46]. (**B**) A schematic illustration of gold nanoparticles-based colorimetric CRISPR assay. Reproduced with permission from Ref. [52].

Colorimetric sensors are popular because they can read the detection results directly with the naked eye, and colorimetric lateral flow assay (LFA) also has many applications in CRISPR/Cas-based detection (Figure 4A). The sample pad, control-line (C-line), and test-line (T-line) of commercial universal test strips were precoated with gold particle-bound anti-FAM antibodies, biotin ligands, and anti-rabbit antibodies, respectively. Biotin- and FAM-modified ssDNA served as reporters for CRISPR/Cas12a-based diagnostics, the reporters were cleaved and captured by T-line when the CRISPR/Cas system was activated, and a positive result was reported. Additionally, a negative result was reported when the reporters were captured by C-line [46]. A similar strategy is used for the on-site detection of *Pseudomonas aeruginosa*, *Leptosphaeria maculans*, and African swine fever virus, with miniaturized equipment and friendly operations flexibly applied in different fields; it is a promising POCT candidate for response to public health events [47,48,49]. Although the LFA-based CRISPR diagnosis is challenging to quantify, its benefits of rapid detection and high sensitivity have significant field application value. Furthermore, LFA sensing methods are also available for Cas9-based diagnostics. After isothermal amplification, the target sequence can be introduced to biotin by biotin-modified primers, while being bound to the avidin-modified T-line. The gold nanoparticles labeled dCas9/crRNA complexes via surface binding ssDNA, which can partially hybridize with crRNA, similar to gold nanoparticle labeled nucleic acid antibodies [50]. Another Cas9-based LFA assay also similarly requires the introduction of special chemical groups (digoxin, biotin, and fluorescein isothiocyanate isomer (FITC)) to the target sequence during isothermal processing using primers with specific chemical modifications. The Cas9nAR specifically selects isothermal amplification sites, and chemically modified primers introduce specific groups to the amplicon that can be captured by the T-line [51].

In addition to LFA, other colorimetric probes have also been used to visualize CRISPR-based detection. The gold nanoparticle solution changes from red to purple after particle aggregation due to its electromagnetic properties, which is a promising CRISPR colorimetric strategy. The ssDNA or ssRNA can be modified on the surface of gold nanoparticles through sulfhydryl groups and prevent the aggregation of nanoparticles. Upon trans-cleavage of ssDNA or ssRNA by Cas12a/13a, gold nanoparticles can aggregate, and the change in solution color can serve as a reporter (Figure 4B) [52,53]. With this strategy, SARS-CoV-2 can be detected and the results are consistent with those of clinical testing [54]. Furthermore, the nanozymes additionally seem to be a valuable probe for visualizing CRISPR-based detection. When combined with the CRISPR/Cas13a system, the nanozyme-based immunosorbent assay (NLSA) can amplify the reporter signal via the catalytic reaction of nanozymes and improve sensitivity, allowing for amplification-free RNA detection [55]. NLSA is a signal amplifier for target RNA detection, it is similar to the enzyme-linked immunosorbent assay (ELISA), which involves adding reagents in steps to a functional surface to catalyze the substrate and produce a color product that can be used to read the signal. NLSA was more sensitive than ELISA because the substrate conversions of nanozymes with catalytic metal particle compositions were higher than the comparable native enzymes. Although colorimetric CRISPR-based diagnosis is challenging to quantify, its benefits of rapid detection and high sensitivity have significant field application value. As shown in Table 3, the colorimetric sensing strategy enables CRISPR diagnosis to achieve a short test time without sacrificing sensitivity, increasing the chances that CRISPR detection will be used in the field.

## 3. Advanced Strategy for CRISPR/Cas-Based POCT

A POCT method should be sensitive, user-friendly, fast, powerful, equipment-independent, and low-cost [20]. The integration of molecular diagnostics into small devices, while avoiding environmental interferences for on-site detection, can effectively reduce patient and hospital burden and improve response efficiency to public health emergencies. In particular, researchers are very interested in introducing microfluidics and smartphones into CRISPR-based diagnostics in order to achieve rapid, sensitive, and accurate POCT.

### 3.1. Diagnostics on a Chip

**Figure 5:**
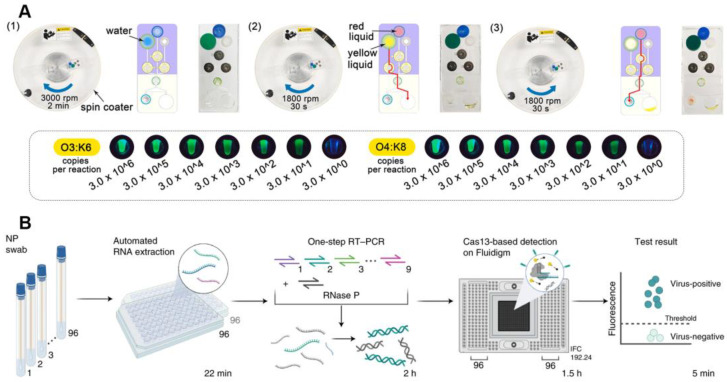
The CRISPR-based detection system on the chip. (**A**) A schematic diagram of CRISPR-based diagnostic system based on a chip. Reproduced with permission from Ref. [56]. (**B**) A multiplexed CRISPR-based microfluidic platform for respiratory-related virus detection. Reproduced with permission from Ref. [64].

Microfluidic chips have the advantage of being integrated, high-throughput, portable, and small in volume, which makes microfluidic-based POCT methods faster; they have higher throughput and are less costly. The rapid (less than 50 min) detection of pathogenic nucleic acids can be achieved by sequentially performing nucleic acid extraction, amplification, and CRISPR-based detection on the microfluidic chip by controlling the fluidic flow through rotary valves. The nucleic acid detection limit of *Vibrio parahaemolyticus* reaches 30 copies/reaction (Figure 5A) [56,57]. To eliminate the requirement for a centrifuge when releasing nucleic acids, Wu et al. further designed an on-chip nucleic acid extraction method, push-pulled by syringe and magnetic beads, that was simple to use and low-cost [58]. On the fluid chip, all test components are kept in advance. It is possible to precisely control the flow and mixing of liquids with the use of a rotating valve and syringe. Within 80 min, DNA extraction, isothermal amplification, and a CRISPR-based assay can be completed. Furthermore, the integrated fully-closed nucleic acid detection based on the microfluidic device can avoid the infection of operators with pathogens while avoiding the contamination of nucleic acid aerosols, providing a promising POCT strategy for coping with urgent public health safety events such as SARS-CoV-2 [59]. Nguyen et al. also developed a paper-based CRISPR sensor for SARS-CoV-2 detection that could be embedded in masks for convenient use in resource-limited areas [60]. Each module needed for CRISPR diagnosis was placed on the outside or inside of the masks. For viral collection, collection pads were placed inside the masks facing the patient’s mouth and nose. The whole set of lyophilized reagents needed for CRISPR diagnostics is contained in μPAD, which are microfluidic paper-based analytical devices that receive any liquid and viral particles gathered from the sample collection pad.

Multiple target sequences are difficult to detect with a single CRISPR-based diagnostic assay, and high-throughput microfluidics provide a multiplexed strategy for CRISPR-based diagnostics [61]. Simple multiplexed chips are usually centrifugation-assisted and symmetrically separated into several separate detection zones, that can be detected without interference with each other, using centrifugal force [62]. Similarly, paper-based microfluidic chips can also be designed to be multiplexed with multiple independent detection modules, and CRISPR reagents against different targets are lyophilized in different regions, enabling diagnosis when the sample solution is chromatographed to the detection region [63]. This paper-based CRISPR microfluidic diagnosis provides an extremely low-cost strategy. In addition, to improve the detection throughput, Ackerman et al. developed combinatorial arrayed reactions (mCARMEN) based on a CRISPR/Cas13a diagnostic system and microfluidics that can detect up to 26 respiratory-related viruses, including coronaviruses such as SARS-CoV-2 and multiple influenza viruses (Figure 5B) [64]. To reduce costs and errors, mCARMEN uses a commercialized fluidic circuit (IFC) tailored to Fluidigm Biomark. IFC can be manually loaded and created to order. Samples in reaction chambers for detection are completely mixed by the Fluidigm controller, which offers fine control over fluid flow on the IFC. Using a customized automated process with a scan time of 1–3 h, the Fluidigm’s fluorescence intensity was quantified as a signal output. The mCARMEN platform is highly scalable and can detect infectious pathogens and mutations using clinical laboratory instrumentation. Simultaneously, the high-throughput capability of this technology has the potential to reduce physical effort, while improving diagnostic efficiency, when dealing with public health emergencies.

### 3.2. Smartphone-Based Portable Detection

**Figure 6:**
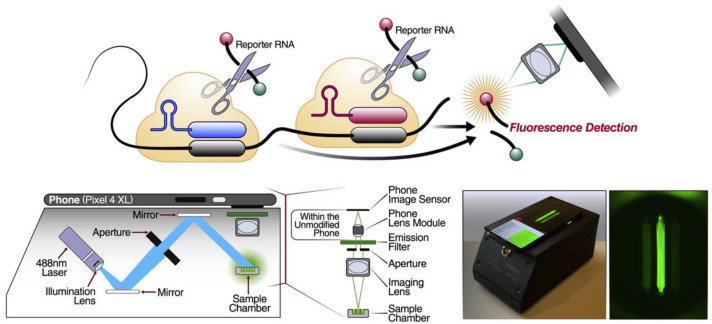
Smartphone assisted portable CRISPR-based detection. A schematic diagram of a mobile phone-based microscope for fluorescence detection after running the Cas13a assay. Reproduced with permission from Ref. [68].

Most CRISPR-based diagnostics rely on fluorescence signals and changes as small as 1% can be sensed by smartphone-based detectors, so a combination of smartphone-based CRISPR diagnostics is a highly sensitive strategy of POCT [65]. The smartphone-based visual detection method can also fully close the detection system without uncapping and avoid nucleic acid aerosol contamination, while maintaining the same positive and negative rates as clinical test results [66]. To determine whether the detection results were positive or negative, the acquired fluorescence images were quantified using custom software equipped with binary classification models [67]. This smart strategy even has the potential to share data with servers to track infectious diseases in real-time, which would be extremely helpful in responding to public health emergencies. More importantly, rather than just qualitative analysis, Fozouni et al. used the smartphone to convert the fluorescence signal into a viral load, providing a potential quantitative strategy for nucleic acid pocketing (Figure 6) [68]. This method offers amplification-free nucleic acid detection since a single target RNA can activate several Cas13a RNPs, substantially doubling the active enzyme concentration, by utilizing multiple RNPs that identify various sections of the same viral target RNA. Instead of specialist equipment, devices that can be produced in large quantities and are adequate to capture the minute fluorescent signal emitted by CRISPR/Cas13a include a custom-built mobile phone fluorescence microscope and reaction chamber. Additionally, this method’s sensitivity is roughly an order of magnitude higher than that of utilizing a plate reader, since customized equipment decreases measurement noise while enabling rapid signal collection.

### 3.3. Functional DNA-Assisted Detection of Non-Nucleic Acid Targets

**Figure 7:**
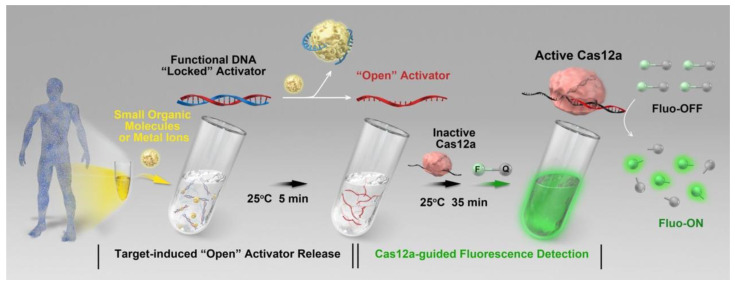
A schematic diagram of functional DNA-assisted CRISPR-based diagnostics for non-nucleic acid target detection. Reproduced with permission from Ref. [70].

The characteristic that ensures the CRISPR/Cas system can only detect nucleic acid molecules (DNA or RNA) limits its application in the diagnosis of non-nucleic acid analytes. It is a flexible strategy to convert non-nucleic acid to nucleic acid signal by using the functional DNA (fDNA, aptamer-based DNA walker) in order to fully exploit the advantages of highly sensitive detection by CRISPR/Cas [69]. The presence of the analytes can cause the functional DNA to be unlocked, which activates the CRISPR/Cas12a and causes trans-cleavage of the fluorescent probe, which can be detected as a fluorescent signal (Figure 7) [70]. The system was made up of an fDNA molecule that was matched to the Cas12a RNP, an ssDNA reporter with fluorescent and quenching groups on both ends (ssDNA-FQ), and Cas12a RNP. The fDNA is in a locked state without the target analyte, which stops Cas12a from initiating trans-cleavage. When the target analyte interacts with the fDNA, Cas12a trans-cleavage activity is activated, resulting in the degradation of the ssDNA-FQ and the generation of a fluorescent signal that can be picked up by a portable fluorometer. The adenosine 5′-triphosphate (ATP) and sodium ions (Na^+^) can be detected at ambient temperature (25 °C) using this method, with a detection limit of 4.75 μM and 0.10 mM. This strategy expanded the detection objects of CRISPR/Cas-based diagnosis to pathogens, cells, exosomes, metal ions, small molecule compounds, and so on, accelerating the development of the CRISPR/Cas-based POCT [70,71,72,73]. Moreover, when a T7 promoter is added to the functional DNA, the in vitro transcription reaction response begins only after the functional DNA has been unlocked by the analytes [74]. An unlocked functional DNA can be transcribed multiple times in vitro, which appears to be a promising approach to further amplify the detection signal. In Table 4, functional DNA-assisted CRISPR methods for different non-nucleic acid target detection are summarized. The functional DNA has a broad variety of target recognition capabilities, therefore these methods could considerably expand the use of the CRISPR-Cas system to many additional targets, opening up a new toolbox for bioanalysis and biomedical research.

## 4. Conclusions and Outlook

Many CRISPR-based diagnostic techniques have been reported over the past few years, and the CRISPR-based POCT has developed rapidly. The potential of CRISPR-based detection as a new generation of POCT method is not diminished despite the fact that there are still some challenges, such as the need to scale up targets using isothermal amplification, the paucity of quantitative analyses, and the absence of established standardized procedures [75]. These constraints are also being overcome. Additionally, a variety of signal sensing techniques may be added to the detection system thanks to the scalability of the CRISPR system, and these techniques can also be compatible with the commercial platform to accomplish process automation and standardization [64]. In terms of both qualitative and quantitative analysis, CRISPR-based POCT has significant potential and versatility, and its customizable and flexible features offer a viable emergency strategy for the detection of acute infectious disorders. At the same time, the SARS-CoV-2 pandemic also led to the development of numerous sophisticated CRISPR-based POCT technologies, which not only assisted with the public health management, but also significantly aided the development of a new generation of POCT [27,64,68].

In this study, we reviewed the sensing methods for CRISPR-based diagnosis and the advanced strategies for CRISPR/Cas-based diagnostic devices, and showed that CRISPR-based POCT can not only achieve an accurate qualitative and quantitative analysis of pathogens, but can also be extended to other fields of non-nucleic acid molecular diagnosis. The majority of these reported diagnostic methods can achieve the rapid, portable, accurate, and sensitive detection of epidemics, providing many candidate platforms for future public health emergencies. Although CRISPR-based POCT tools have made ground-breaking strides, it is complex to translate cutting-edge technologies into usable applications, and there are few commercially accessible kits or clinically useful devices. Future work may need to focus more on convenience, multiplexing, and the calibrability of diagnostic methods, as well as sensing methods that can provide more standardized patterns of signal processing.

## Figures and Tables

**Table 1:** An overview of key features of each class 2 CRISPR system.

Features	Type			
	Cas9	Cas12a	Cas13a	Cas14
PAM	NGG	TTTN	-	-
Target	dsDNA	(ds/ss)DNA	ssRNA	ssDNA
Trans-cleavage	No	Yes	Yes	Yes
Trans-cleavage substrates	-	ssDNA	ssRNA	ssDNA

**Table 2:** An overview of performance of CRISPR detection based on electrochemical signal sensing.

Method	Target	Test Time	Sensitivity	Reference
E-CRISPR	Viral nucleic acids (HPV-16 and PB-19)	30 min	50 pM	[39]
CRISPR/Cas12a-Mediated Electrochemical Nucleic Acid Sensing	DNA	60 min	30 pM	[40]
E-DNA Sensor	DNA	60 min	10 fM	[41]
CRISPR/Cas13a-Powered Electrochemical Microfluidic Biosensor	microRNA	4 h	10 pM	[42]
Label-free Impedance Biosensing	bacterial DNA (*E. coli* and *S. aureus*)	1.5 h	3 nM	[43]
Electrochemical Strategy for Low-Cost Viral Detection	Viral nucleic acids (HIV and HPV)	3 h	10^4^ copies	[44]
RAA-based E-CRISPR	bacterial DNA (*L. monocytogenes*)	1 h	26 CFU mL^−1^	[45]

**Table 3:** An overview of the performance of CRISPR detection based on colorimetric signal sensing.

Method	Target	Test Time	Sensitivity	Reference
RAA-Cas12a based system (CORDS)	African swine fever virus (ASFV)	60 min	1 fM	[46]
RPA-CRISPR/Cas12a system	*L. maculans*	45 min	4.7 copies per test	[48]
CRISPR/Cas12a-LFD	ASFV	60 min	20 copies per test	[49]
CRISPR/Cas9-mediated Lateral flow nucleic acid assay (CASLFA)	*L. monocytogenes*, genetically modified organisms (GMOs), ASFV	60 min	10^2^ copies per test	[50]
Lateral flow strip combined with Cas9	double food-borne pathogens	3 h	10^2^ CFU mL^−1^	[51]
Combines the Cas12a with universal AuNPs strand-displacement probe	SARS-CoV-2	20 min	2.7 × 10^2^ CFU mL^−1^	[53]
RT-RPA-coupled CRISPR/Cas12a colorimetric assay	SARS-CoV-2	60 min	1 copy per test	[54]

**Table 4:** An overview of functional DNA-assisted CRISPR methods for different non-nucleic acid target detection.

Method	Target	Signal Readout	Sensitivity	Reference
DNAzyme walkers-triggered CRISPR assay	serum amyloid A-1 protein (SAA1) and coagulation factor V (FV)	Fluorescent	30.00 pg mL^−1^ for SAA1 and 200.00 pg mL^−1^ for FV	[69]
Functional DNA Regulated CRISPR-Cas12a Sensor	ATP and Na^+^	Fluorescent	4.75 μM for ATP and 0.10 mM for Na^+^	[70]
MDANs-Cas12a	circulating tumor cells (CTCs)	Fluorescent	26 cells mL^–1^	[72]
CRISPR/Cas12a	exosome	Fluorescent	10^3^ particles μL^−1^	[73]
APC-Cas	*Salmonella* Enteritidis cells	Fluorescent	1 CFU per test	[74]

## Data Availability

Not applicable.
